# Using the Amino Acid Network to Modulate the Hydrolytic Activity of β-Glycosidases

**DOI:** 10.1371/journal.pone.0167978

**Published:** 2016-12-09

**Authors:** Fábio K. Tamaki, Diorge P. Souza, Valquiria P. Souza, Cecilia M. Ikegami, Chuck S. Farah, Sandro R. Marana

**Affiliations:** Departamento de Bioquímica, Instituto de Química, Universidade de São Paulo, São Paulo, Brazil; Russian Academy of Medical Sciences, RUSSIAN FEDERATION

## Abstract

The active site residues in GH1 β-glycosidases are compartmentalized into 3 functional regions, involved in catalysis or binding of glycone and aglycone motifs from substrate. However, it still remains unclear how residues outside the active site modulate the enzymatic activity. To tackle this question, we solved the crystal structure of the GH1 β-glycosidase from *Spodoptera frugiperda* (Sfβgly) to systematically map its residue contact network and correlate effects of mutations within and outside the active site. External mutations neighbouring the functional residues involved in catalysis and glycone-binding are deleterious, whereas mutations neighbouring the aglycone-binding site are less detrimental or even beneficial. The large dataset of new and previously characterized Sfβgly mutants supports that external perturbations are coherently transmitted to active site residues possibly through contacts and specifically disturb functional regions they interact to, reproducing the effects observed for direct mutations of functional residues. This allowed us to suggest that positions related to the aglycone-binding site are preferential targets for introduction of mutations aiming to further improve the hydrolytic activity of β–glycosidases.

## Introduction

The deeper understanding of the molecular functioning of enzymes has allowed us to exploit their applications as biotechnological tools. For instance, the exploitation of biomass-degrading enzymes for the production of soluble sugars from cellulose has contributed to the production of second generation biofuels [1]. In this process, cellulases processively degrade cellulose chains to cellobiose, which is further hydrolysed to glucose by β-glycosidases. The use of more efficient β-glycosidases simultaneously improves glucose production and decreases cellulase retro inhibition by its product [2]. In this sense our ability to improve the efficiency of degrading enzymes (e.g. cellulases and β-glycosidases) is one of the main challenges that need to be overcome in order to achieve economic viability for biofuel production from cellulose.

The β-glycosidase from the fall armyworm *Spodoptera frugiperda* (Sfβgly) is an extensively studied member of glycoside hydrolase family 1 (GH1) [3,4] and is an attractive template from which to gain insight on how to rationally design more efficient enzymes. The Sfβgly catalytic mechanism relies on a pair of glutamates, the proton donor E187 and the nucleophile E399 [5], which are responsible for glycosidic bond cleavage in a double substitution mechanism [3]. Residues R97 and Y331 are residues also described as catalysis-related (CR) [6] because they modulate the p*K*_a_ of catalytic glutamates E187 and E399. In addition to residues involved in catalysis, the active site is also composed of two sets of residues involved in the substrate binding: the highly conserved residues in the glycone-binding site (GBS) [7–10] interact to the substrate glycone through hydrogen bonds and stacking interactions, while residues lining the less-conserved aglycone-binding site (ABS) [9,11,12] modulate the Sfβgly transglycosilase activity [13] and its preference for alkyl or glucose moieties [10].

Recent studies have expanded our understanding on how mutations external to the Sfβgly active site affect its hydrolytic activity. For instance, single mutations distant from the active site modulate Sfβgly substrate specificity [11], while mutations of covariant positions surrounding its active site are deleterious [14]. These data suggest that mutational effects can be transmitted from external to functional active site residues [9]. However, despite the existence of a massive kinetic dataset for mutations outside the active site, the lack of high-resolution structural data for Sfβgly has hindered an accurate model to map contacts among residues from the functional regions (CR, GBS and ABS) and its neighbours.

In this paper we present the Sfβgly crystal structure (PDB ID 5CG0), which was used to systematically identify amino acids directly and indirectly contacting the functional residues composing the CR, GBS and ABS in Sfβgly. The exploitation of this residue contact network allowed us to accurately correlate the effects of mutations from within and outside the active site. The kinetic data for the hydrolysis of two synthetic substrates confirm that effects observed for mutations of active site residues are coherently reproduced by mutations of their corresponding contacting neighbouring residues. We also present the kinetic data for one new active site mutant (R97) and 8 new external mutants that corroborate the concerted modulation of Sfβgly hydrolytic activity according to the contacted functional residues. Five of these mutants contact the ABS and enhance the hydrolysis rate of synthetic substrates by Sfβgly, of which two also enhance the hydrolysis of cellobiose. Given that mutations enhancing Sfβgly hydrolytic rates are overwhelmingly located in the ABS and at its neighbourhood, our analysis could help suggesting new hotspots for the introduction of variability aimed at producing more efficient β-glycosidases. The data here presented support that the contact network coherently transmit the mutational information between the active site residues and their neighbours, playing a central role in fine-tuning Sfβgly hydrolytic activity. The general principle here proposed seems to be universal for GH1 β-glycosidases and could be applied to rationally design more efficient enzymes towards the economically viable production of cellulosic biofuel.

## Experimental Procedures

### Mutagenesis, expression in *E*. *coli* and purification of wild-type Sfβgly and its mutants

The wild-type Sfβgly gene cloned into pAE plasmid [15] was used as template for mutagenic PCR reactions using mutagenic primers (Table A in S1 File) and the QuikChange site-directed mutagenesis kit (Stratagene, La Jolla, CA, USA), following the manufacturer’s instructions. The introduction of mutations was confirmed by DNA sequencing. Wild-type or mutants of Sfβgly cloned into pAE were used to transform NovaBlue (DE3) competent *E*. *coli* cells (EMD Millipore, Billerica, MA, USA).

Transformed bacteria were subsequently plated on LB-agar containing 50 μg/mL ampicillin and grown overnight at 37°C. Individual colonies were used to inoculate LB broth containing 50 μg/mL ampicillin and grown to an optical density of 0.5 at 600 nm, when 0.4 mM isopropyl β-D-1-thiogalactopyranoside was added to induce the expression of recombinant wild-type and mutants of Sfβgly for 16 h at 20°C. Cells expressing recombinant enzymes were collected by centrifugation at 4,000 × g for 20 min (4°C) and frozen at -80°C until use. Thawed cells were resuspended (100 mM sodium phosphate pH 7.4 containing 200 mM NaCl, 60 mM imidazole and 10% (v/v) glycerol) and sonicated using a Branson Sonifier 250 adapted with a microtip (three 15 s ultrasound pulses, output 3, with 1 min intervals in ice to avoid heat denaturation). The lysate was centrifuged (13,200 × g, 20 min, 4°C) and Sfβgly and its mutants were purified from the soluble fraction by batch affinity binding using 200 μL Ni-NTA Agarose resin (4°C, 1 h) (Qiagen, Hilden, Germany). After pelleting the resin and washing it 5 times (100 mM sodium citrate—sodium phosphate pH 6.0 containing 200 mM NaCl and 60 mM imidazole), recombinant Sfβgly was eluted by the addition of 500 mM imidazole in the same buffer. The purity of each Sfβgly sample was verified using SDS-PAGE [16]. Purified enzymes underwent desalting in minitrap G-25 column using 100 mM sodium citrate—sodium phosphate pH 6.0 (GE Healthcare, Upsala, Sweden) and were stored at 4°C. Protein concentration was spectrophotometrically determined at 280 nm using 6 M guanidinium hydrochloride in 20 mM sodium phosphate pH 6.0. The theoretical extinction coefficients (Ɛ_280nm_) were calculated from the primary sequences of wild-type and mutants of Sfβgly using the ProtParam server (http://web.expasy.org/protparam/).

### Expression of wild-type Sfβgly in *Pichia pastoris* and purification for crystallization

Because recombinant wild-type Sfβgly expressed in *E*. *coli* (see above) produced poor quality crystals (data not shown), it was expressed in *Pichia pastoris* [13] for further tests. The DNA sequence coding for wild-type Sfβgly was cloned into the pPIC9 plasmid and used to transform *P*. *pastoris* GS115 competent cells (Life Technologies) following the manufacturer’s instructions. The resulting construct expresses the full-length protein plus an N-terminal signal peptide that directs protein secretion and concomitant removal of the signal peptide [13]. The secreted recombinant protein is therefore expected to consist of native Sfβgly (residues 21–509), lacking the first 20 amino acids corresponding to the signal peptide. Colonies expressing recombinant Sfβgly were selected using minimal media MD-agar plates containing 1% (w/v) ammonium sulfate, 2% (w/v) glucose and 4 × 10^−5^% (w/v) biotin. Colonies were subsequently grown in YPD [1% (w/v) yeast extract, 2% (w/v) peptone and 2% (w/v) glucose] and then transferred to BMM [100 mM potassium phosphate pH 6.0 containing 1.34% (w/v) YNB, 4 × 10^−5^% (w/v) biotin and 1% (v/v) methanol] for Sfβgly expression for up to 14 days with daily supplementation of 0.1% (v/v) methanol. Cells expressing wild-type Sfβgly were sedimented by centrifugation (3,000 × g, 5 min at 4°C) and the culture supernatant containing Sfβgly was concentrated. The buffer was exchanged to 20 mM Tris-HCl pH 7.5 using tangential filtration in a hollow fiber cartridge system (10,000 NMWC) (GE Lifesciences, Westborough, MA, USA). Concentrated Sfβgly was purified using a Mono Q HR 5/5 column coupled to an ÅKTA FPLC system (GE Lifesciences, Uppsala, Sweden) as previously described [6]. Purified Sfβgly was concentrated by reverse dialysis using PEG 35,000 to 3.5 mg/mL and crystallized by vapour-diffusion in sitting-drop plates using a solution of 100 mM Bis-Tris-HCl pH 6.3 containing 100 mM NaCl and 22% (w/v) PEG 3350.

### Crystallographic data collection, structure determination and refinement

Crystallographic data were collected using a microfocus copper rotating anode Rigaku MicroMax-007HF X-ray generator (radiation wavelength 1.54 Å) and an R-AXIS IV++ image plate X-ray detector at the Instituto de Química of the Universidade de São Paulo. Crystals were cooled directly in a 100 K nitrogen-gas steam and data were collected using 0.3° oscillation per frame (1200 frames). Collected data were processed in space group P1 using HKL2000 [17]. Initial phases for Sfβgly were obtained by molecular replacement using the program Phaser [18] from the CCP4 package (Collaborative Computational Project, Number 4, 1994) and the structure of the β-glycosidase from *Neotermes koshunensis* (PDB ID 3VIF) [19] as the search model (46.18% identity with Sfβgly). The unit cell contains six Sfβgly molecules. Cycles of structure refinement were carried out using the REFMAC5 program [20] from the CCP4 suit and iteratively refined in real space with WinCoot 0.7.2.1 [21]. Water molecules were added automatically and checked by manual inspection while Tris(hydroxymethyl)aminomethane (Tris) molecules and N-acetyl-D-glucosamine (NAG) groups were manually fitted followed by subsequent rounds of automated refinement. The refined structure underwent validation using PROCHECK [22], MolProbity [23], Rampage [24] and tools available in WinCoot. Distances smaller than 5 Å between atoms of different residues are here considered as one contact, as previously adopted [25]. Structures were visualized using the PyMOL molecular graphics system v1.1 (Schrödinger, LLC) and the DeepView/SwissPDBViewer v3.7 software [26]. PyMOL was also used for structural alignments, from which we used the resulting RMSD values for comparisons among different structures. Atomic coordinates and structure factors of the Sfβgly crystal structure have been deposited in the Protein Databank (www.pdb.org) with the accession code 5CG0.

### Kinetic characterization of wild-type Sfβgly and its mutants

The kinetic parameters (*k*_cat_ and *K*_m_) of purified wild-type Sfβgly and mutant enzymes were determined by measuring the initial rates (*v*_*0*_) of cellobiose, *p*-nitrophenyl-β-D-glucopyranoside (NPβglc) and *p*-nitrophenyl-β-D-fucopyranoside (NPβfuc) (Sigma, St. Louis, MO, USA) hydrolysis at 30°C using at least 10 different substrate concentrations prepared in 100 mM sodium citrate–sodium phosphate pH 6.0. The rate of cellobiose hydrolysis was performed as previously described [27], and the hydrolysis of synthetic substrates NPβglc and NPβfuc was detected following the absorbance at 420 nm from the release of *p*-nitrophenolate after addition of 250 mM sodium carbonate–sodium bicarbonate pH 11.0. Relative volume of reaction:stop solution was 1:10. The fitting of *v*_*0*_ and [S] to the Michaelis-Menten equation using the Enzfitter software (Elsevier-Biosoft, Cambridge, UK) allowed the determination of kinetic parameters (*K*_m_ and *k*_cat_) and errors. Data from mutants previously studied were collected from the literature.

We compared the relative *k*_cat_/*K*_m_ (catalytic efficiency) for the hydrolysis of different substrates and the calculated mutational effects are present as the ratio between the catalytic efficiency of mutants and wild-type Sfβgly [(*k*_cat_/*K*_m_)_mut_/(*k*_cat_/*K*_m_)_WT_]. While negative changes present ratios between 0 and 1, positive effects yield ratios higher than 1. Unless otherwise stated, all mutants tested here are correctly folded and present structural features similar to the wild-type enzyme, as seen by tryptophan fluorescence spectra or thermal shifting assays [9,14,28].

### Sfβgly sequence analysis

A multiple sequence alignment of GH1 β-glycosidases containing 1551 non-redundant sequences (identity lower than 80%) was used as input for DCA analysis (DCA webserver) [29] and for a weblogo creation (http://weblogo.threeplusone.com/) [30]. The DCA resulted in 111,156 residue-residue pairings with absolute values for Direct Information (DI) ranging from 0.4458874 to 6.28e^-5^. We manually analysed the top 1000 contacts (<1%, DI ranging from 0.4458874 to 0.0261080).

## Results and Discussion

### Structural features of Sfβgly and its active site

**Fig 1:**
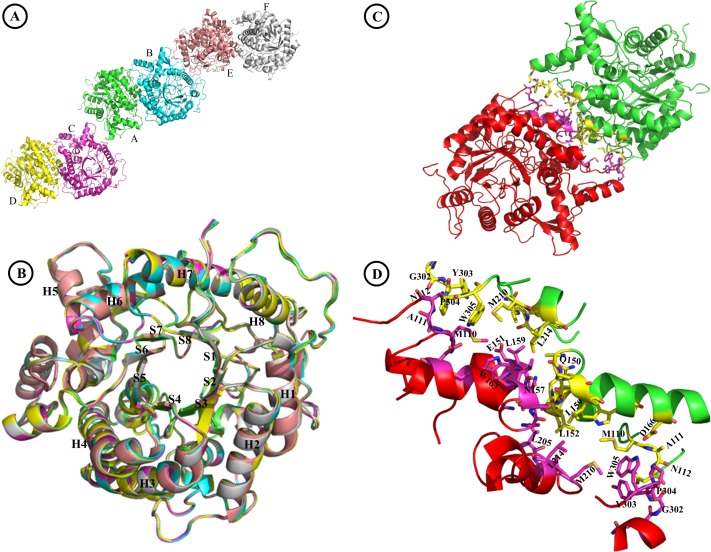
Crystallographic structure of Sfβgly (PDB ID 5CG0). **A**–Six Sfβgly chains (A-F) are observed in the asymmetric unit; **B**–Superposition of the six Sfβgly refined chains (A-F). The (β/α)_8_ secondary structures are labeled; **C**–Cartoon representation of the Sfβgly crystallographic dimer (Chain A: Green; Chain B: Red) **D**–Detailed interaction surface of a Sfβgly dimer. Residues in the dimerization interface are coloured yellow (Chain A) and pink (Chain B).

We have solved the crystal structure of the Sfβgly (PDB ID 5CG0) to systematically map its contact network and for the further accurate correlation between effects of mutations within and outside the active site. To obtain diffraction-quality crystals, the full-length recombinant wild-type Sfβgly lacking the signal peptide (residues 21–509) was expressed as a secreted protein in *P*. *pastoris* and purified by ion-exchange chromatography [13]. Sfβgly crystals belong to space group P1, with 6 molecules (chains A-F) in the asymmetric unit (Fig 1A). The statistics of data collection, refinement and quality of the final structure are shown in Table B in S1 File. Chain A was used as the representative Sfβgly monomer since it presents the lowest atomic B-factors and best fit of electron density for the greatest number of amino acid residues (488 out of 489) (Table B in S1 File). On the other hand, chain F presents the relatively higher average B-factor and lacks electron density for residues 472 and 495–509. Although chains A, B and C present the best quality parameters, the six chains are very similar, as seen by their structural alignment (Fig 1B) and RMSD values for aligned atoms (ranging from 0.084 to 0.192 Å, Table C in S1 File). Those values are much lower than the one obtained for the Sfβgly structural model generated by homology [14] (Table C in S1 File) and when compared to RMSD values obtained by aligning the Sfβgly (Chain A) to homologous β-glycosidases from PDB (ranging from 0.571 to 1.046 Å, Table D in S1 File). This confirms that solving the Sfβgly crystal structure was fundamental to accurately measure distances among its residue atoms and thus the determination of its structural network. Sfβgly presents the typical (β/α)_8_ barrel fold seen for GH1 enzymes (Fig 1B) [3,4,19,31,32]. The recombinant expression of Sfβgly in *P*. *pastoris* yielded a protein glycosylated at N206. We were able to model two NAG moieties at this position in chains A, B and D and only one NAG at this position in chains C, E and F, for a total of nine NAG residues.

Sfβgly chains A-B, C-D and E-F form dimers in which the monomers are related by non-crystallographic pseudo-twofold symmetry (Fig 1C and Figure A in S1 Fig). The dimerization interfaces involve: i) residues 110–112, 166 and 210 from one chain contacting residues 301–305 of its partner; and ii) residues 150–153, 156–159 and 163 from one chain contacting residues 205, 207, 211 and 214 from the other chain (Fig 1D). These interactions are reciprocal between Sfβgly chains forming dimers. So, as an example, residue D166 from chain A interacts to residues P304 and W305 form chain B, and D166 from chain B interacts to P304 and W305 from chain A. PDBePISA [33] predicts that this crystallographic dimer interface is stable with a maximum interface score value of 1 for complex formation (interface area: 905 Å; Solvation energy gain on complex formation: - 8.7 kcal/mol). Sfβgly dimerization is consistent with previous data observed in size exclusion chromatography, which reveals a rapid equilibrium between monomers and dimers that depends on the ionic strength [34]. The crystal structures of GH1 *N*. *koshunensis* β-glycosidase (PDB ID 3AHZ, Figures B-C in S1 Fig) [19] and *Brevicoryne brassicae* myrosinase (PDB ID 1WCG, Figure D in S1 Fig) [35] also exhibit the same dimeric interface observed for Sfβgly. Moreover, Direct Coupling Analysis (DCA) [29] infers statistical co-evolutionary coupling between Sfβgly residue pairs at the protein surface involved in dimeric contacts: 110–152, 112–150, 111–163, 152–163, 157–163 and 156–211 (Table E in S1 File), supporting that β-glycosidase dimerization is a common, but previously overlooked, feature among insect GH1 enzymes. Therefore, all those data combined would suggest that this observed dimer interface is not a crystallographic artefact and could be relevant for β-glycosidases in solution at high concentrations. On the other hand, no evidence of cooperativity or allostery has been observed for the Sfβgly hydrolytic activity during enzymatic assays, which employ low enzyme concentrations (a few micromolar). Consistent with this, the mutation N112A present at the Sfβgly dimerization interface predicted by DCA [29] and PDBePISA [33] has no significant effect on its kinetic parameters when compared to the wild-type enzyme [14].

**Fig 2:**
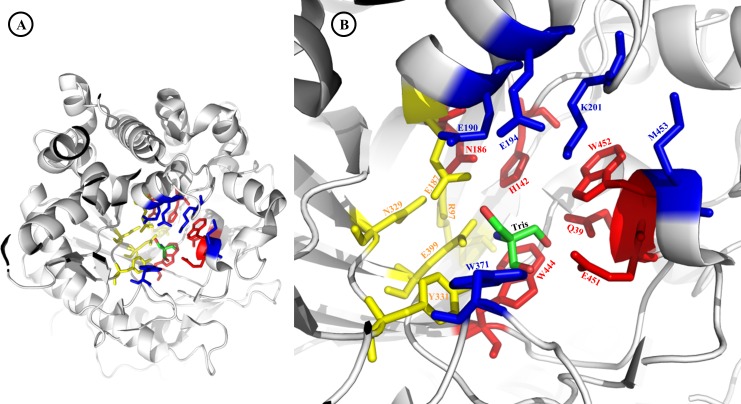
Sfβgly active site. **A**–Overview of Sfβgly active site. Note that ABS residues are located at the active site entrance, whereas the GBS and CR residues are located at the bottom of the active site pocket. **B**–Detailed view of functional residues (in sticks): GBS (red), ABS (blue) and CR (yellow). One Tris molecule (green sticks) is bound to the Sfβgly active site (PDB ID 5CG0).

The refined Sfβgly structure presents a Tris molecule, a known β-glycosidase competitive inhibitor [32, 36, 37] bound at its active site (Fig 2). Substrate access to the active site is achieved by way of an opening at the top of the β-barrel (Fig 2 and S2 Fig) [38]. The oxygen atoms of the carboxylate groups in the side chains of catalytic glutamates E399 and E187 are 3.9 to 4.8 Å apart from each other (as expected for retaining β-glycosidases) and hydrogen bond the Tris molecule complexed at the active site. Moreover, these side chains are less than 5 Å from R97, Y331 and N329, residues involved in p*K*_a_ modulation of the catalytic glutamates [6]. The N329 side chain hydrogen bonds both E187 and E399 side chains, indicating its importance for catalysis. Therefore, the catalytic glutamates E187 and E399 as well as the residues modulating their properties (R97, Y331 and N329) delimit the CR functional region (Fig 2, yellow).

**Fig 3:**
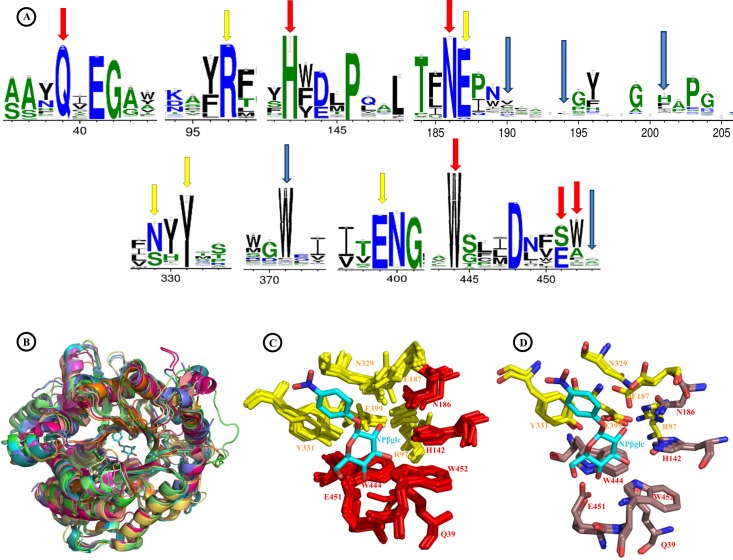
Degree of conservation among β-glycosidases. **A**–Weblogo of active site positions generated from a multiple sequence alignment of 1551 β-glycosidases sequences. Note that residues in ABS positions 190, 194, 201 and 453 (blue arrows) are poorly conserved when compared to GBS (red arrows) and CR (yellow arrows) positions. **B**–Superposition of Sfβgly (chain A) with homologous GH1 β-glycosidases from PDB: 3AI0, 1E6S, 1E4I, 1E56, 1UG6, 2ZOX, 1V03 and 1VFF. **C**–Multiple superposition of active site conserved residues (GBS: red; CR: yellow) from Sfβgly and β-glycosidases shown in (B). The complexed NPβglc (cyan) is from structure 3AI0. The numbering shown is from Sfβgly. **D**–Detailed superposition of active site residues from Sfβgly and 3AI0 (GBS: brown; CR: yellow), demonstrating their similar relative positioning to NPβglc (Cyan).

The Sfβgly active site also has 2 regions associated with substrate binding. The highly conserved glycone-binding site (GBS) is composed of residues Q39, H142, N186, W444, E451 and W452 (Fig 2, red), which hydrogen bond hydroxyl groups from the substrate monosaccharide [7, 32] and are located at the bottom of the active site (S2 Fig, red). The aglycone-binding site (ABS) is composed of residues E190, E194, K201, W371 and M453 (Fig 2, blue) [11], which are located at the entrance (top opening) of the active site (S2 Fig, blue). The ABS is structurally less conserved than GBS, which is illustrated by their amino acid conservation profiles shown in Fig 3A [12,32] and by the variable the length of loop 200–205, which is significantly longer in Sfβgly than in plant β-glycosidases [11]. Besides these minor structural differences among the active sites of several GH1 β-glycosidases, their overall structures and topologies are highly conserved (Fig 3B), especially regarding the atomic positioning of CR and GBS residues (Fig 3C and 3D). Given the high structural similarity of active sites from Sfβgly and from *N*. *koshunensis* β-glycosidase (PDB IDs: 3AI0, complexed to NPβglc, Fig 3D; 3VIK, complexed to cellobiose, S3 Fig) [19], we used their superposition to analyse the fitting of substrates into the Sfβgly active site (Fig 3D; S3 Fig) and correlate structural and mutational data.

### Residues surrounding the active site modulate the Sfβgly hydrolytic activity through the amino acid network

**Fig 4:**
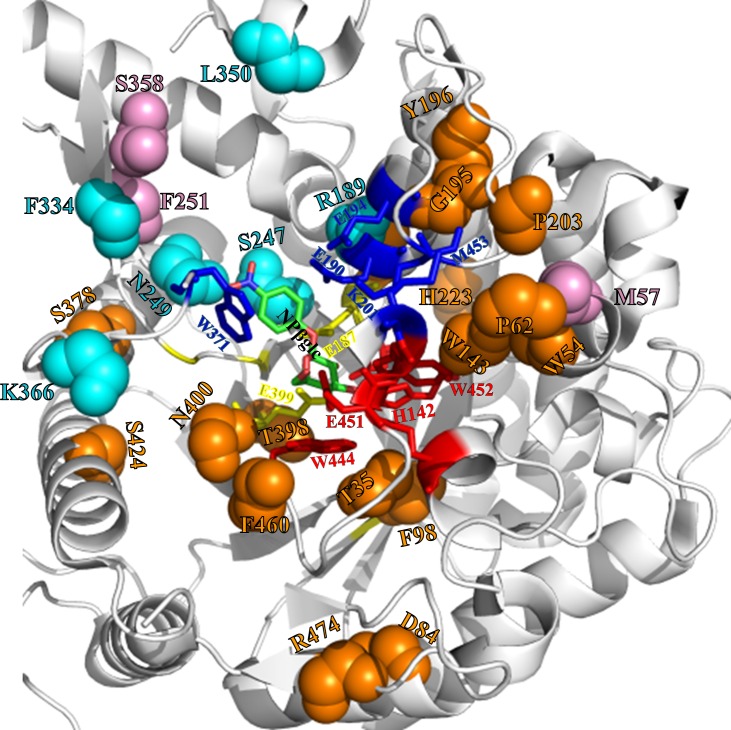
Spatial distribution of mutational effects outside the Sfβgly active site. Highlighted are the main chain (spheres) of amino acids whose mutations cause deleterious (decreases higher than 4-fold using NPβglc, dark orange spheres) and mild decreases (smaller than 4x, light purple spheres) or positive effects (cyan spheres) on Sfβgly activity. Residues outside the active site are labelled with their corresponding colour, as described above. Active site residues from GBS (red sticks), ABS (dark blue sticks) and CR (yellow sticks) are also labelled with their corresponding colour. A substrate molecule, *p*-nitrophenyl β-glycoside (NPβglc), is placed in the active site (green sticks). Note that positive mutations (cyan spheres), are located close to the ABS but not to the GBS.

**Fig 5:**
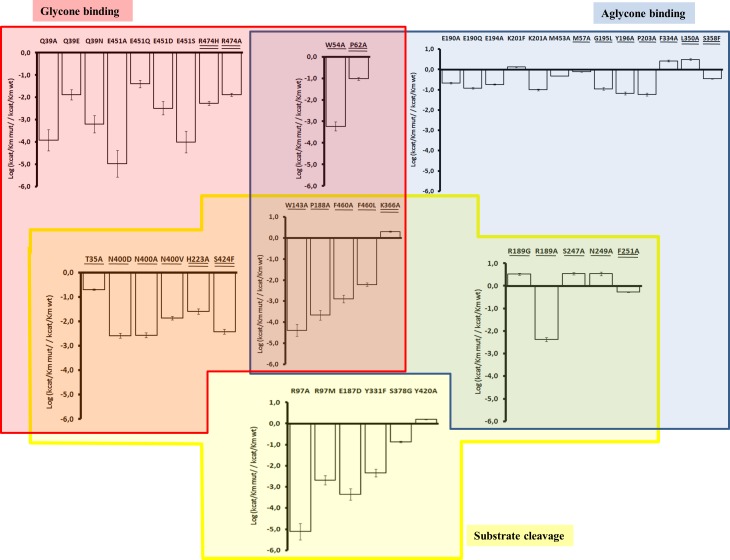
Effects of single mutations on the Sfβgly hydrolytic activity. Mutational effects are shown as the Log of relative catalytic efficiency [(*k*_cat_/*K*_m_)_mut_/(*k*_cat_/*K*_m_)_WT_]. Charts present the same scale for comparisons among mutational effect amplitudes. Mutations are grouped according to the active site residues they are associated. **Red box:** residues associated to the GBS; **Blue box:** positions associated to the ABS; **Yellow box:** residues related to the CR. Residues at L1 (underlined) and L2 (double underlined) associated to more than one functional group (GBS, ABS or CR) are placed in the group intersections. Mutations of active site residues are presented not underlined. Grouping is based on the shortest pathway linking the mutation to the functional region. Four mutants are inactive and not included here: D84A (L2 amino acid contacting GBS and CR residues), W452A (located in the GBS), F98A and T398A (both L1 positions contacting CR residues).

The resolution of the Sfβgly crystal structure allowed us to correlate the structural features of its active site to the large set of enzyme kinetics data that is available for Sfβgly and mutants. Fifty-one single mutations (including 9 new characterized ones) distributed across 37 positions of Sfβgly were analysed (Fig 4). Table 1 presents the effects on the *k*_cat_/*K*_m_ due to mutation of residues involved in the three functional regions of the Sfβgly active site. The logarithmic plot of *k*_cat_/*K*_m_ values also allows a good comparison among amplitudes of mutational effects (Fig 5). Mutant enzymes were characterized using NPβglc as substrate, except for E187D, which due to its very low activity was studied using MUβglc. Mutations of GBS residues Q39, E451 and W452 severely diminish or abolish the hydrolysis of NPβglc (Table 1; Fig 5) because they simultaneously cause a decrease in the affinity for the substrate (increases in *K*_m_) and in the catalytic rates (*k*_cat_) (Table G in S1 File). Similar results have also been previously observed using NPβfuc as substrate [7,14,38] (Table F in S1 File). This marked intolerance of the GBS to perturbations correlates with its high degree of structural conservation among GH1 β-glycosidases (Fig 3A, red arrows, and Fig 3C and 3D). Mutations of CR residues R97, E187 and Y331 also have deleterious effects on Sfβgly hydrolytic activity [6,9]. It is worth noting that the deleterious mutation Y331F in fact positively modulate the enzyme affinity for NPβglc (decrease the *K*_m_); however, the deleterious nature of mutations in CR results from a strong negative modulation of the *k*_cat_ (Table G in S1 File) observed for all CR mutants, which is consistent with the role played by these residues to the enzymatic catalysis. On the other hand, mutations in the ABS residues E190, E194, K201 and M453 result in smaller decreases of the hydrolysis rate of NPβglc (2- to 10-fold) [11], while the mutation K201F increases the Sfβgly hydrolytic activity. Overall, mutations of ABS residues have smooth effects on both *k*_cat_ and *K*_m_. The similar trends observed for all Sfβgly mutants using two synthetic substrates (NPβglc, Table 1; NPβfuc, Table F in S1 File) confirm the beneficial or deleterious nature of each mutation. The tolerance of the ABS to perturbations correlates with its low degree of structural conservation among GH1 β-glycosidases (Fig 3A, blue arrows). This plasticity could be the result of selection pressures over the β-glycosidases impressed by the high diversity of aglycones forming the β-glycosides found in nature. As a practical example of the ABS plasticity, it was verified in the β-glycosidase from *Thermotoga neapolitana* that the double mutant N221S/P342L (mutations of residues associated to the ABS) increases its activity towards the hydrolysis of quercetin-3-glucosides but has neutral effects using the synthetic substrate NPβglc [39].

**Table 1:** Effects on the *k*_cat_/*K*_m_ for the hydrolysis of NPβglc due to mutation of residues involved in the three functional regions of the Sfβgly active site.

Glycone Binding (GBS)		Aglycone Binding (ABS)		Substrate Cleavage (CR)	
Mutation	Relative *k*_cat_/*K*_m_	Mutation	Relative *k*_cat_/*K*_m_	Mutation	Relative *k*_cat_/*K*_m_
T35A^1^	0.208	W54A^4^	0.022	T35A^1^	0.208
*Q39A*^2^	0.00012	M57A^4^	0.765	D84A	inactive
*Q39E*^3^	0.013	P62A^4^	0.094	*R97A*	0.0000008
*Q39N*^3^	0.00063	W143A^4^	0.00036	*R97M*^1^	0.021
W54A^4^	0.022	P188A^4^	0.00021	F98A^4^	inactive
P62A^4^	0.094	R189G^*1*^	3.3	W143A^4^	0.00036
D84A	inactive	R189A^*1*^	0.004	*E187D*^6*^	0.00044
W143A^4^	0.00036	*E190A*^5^	0.213	P188A^*4*^	0.00021
P188A^4^	0.00021	*E190Q*^5^	0.120	R189G^*1*^	3.3
H223A^*2*^	0.026	*E194A*^5^	0.184	R189A^*1*^	0.004
K366A	2.0	G195L^4^	0.112	H223A^*4*^	0.026
N400D^1^	0.00265	Y196A^4^	0.066	S247A	3.5
N400A^1^	0.0027	*K201A*^5^	0.10	N249A	3.5
N400V^1^	0.014	*K201F*^5^	1.3	F251A	0.538
S424F^1^	0.0038	P203A^4^	0.059	*Y331F*^1^	0.016
*E451A*^2^	0.000011	S247A	3.5	K366A	2.0
*E451Q*^3^	0.0404	N249A	3.5	S378G^1^	0.134
*E451D*^3^	0.0033	F251A	0.538	T398A^4^	inactive
*E451S*^3^	0.000101	F334A	2.6	N400D^1^	0.00265
*W452A*^4^	inactive	L350A	3.2	N400A^1^	0.0027
F460A^4^	0.0013	S358F^1^	0.358	N400V^1^	0.014
F460L^1^	0.006	S358A^1^	0.569	Y420A	1.6
R474H^1^	0.0054	K366A	2.0	S424F^1^	0.0038
R474A^1^	0.013	*M453A*^5^	0.48		
		F460A^4^	0.0013		
		F460L^1^	0.006		

Relative *k*_cat_/*K*_m_ corresponds to [(*k*_cat_/*K*_m_)_mut_/(*k*_cat_/*K*_m_)_WT_]. Mutations that cause relative *k*_cat_/*K*_m_ decrement higher or lower than 4 fold are respectively marked in orange and light purple; mutations causing relative *k*_cat_/*K*_m_ increment are in green. Residues belonging to layer 1 are underlined; residues from layer 2 are double underlined; active site residues are in italics. Kinetics from mutations D84A, R97A, S247A, N249A, F251A, F334A, L350A, K366A and Y420A are new data presented here. Remaining kinetic data collected from 1: Mendonça and Marana, 2011 [9]; 2: Marana *et al*., 2002 [7]; 3: Marana *et al*., 2004 [8]; 4: Tamaki *et al*., 2014 [14]; 5: Mendonça and Marana, 2008 [11]; 6: Marana *et al*., 2003 [6]. For calculation of Relative *k*_cat_/*K*_m_, each *k*_cat_/*K*_m mut_ was compared to the *k*_cat_/*K*_mWT_ data presented on the same manuscript in which the mutant enzyme was firstly described: 1: Mendonça and Marana, 2011 [9]; 2: Marana *et al*., 2002 [7]; 3: Marana *et al*., 2004 [8]; 4: Tamaki *et al*., 2014 [14]; 5: Mendonça and Marana, 2008 [11]; 6: Marana *et al*., 2003 [6] (*: This mutant was studied using MUβglc as substrate).

**Fig 6:**
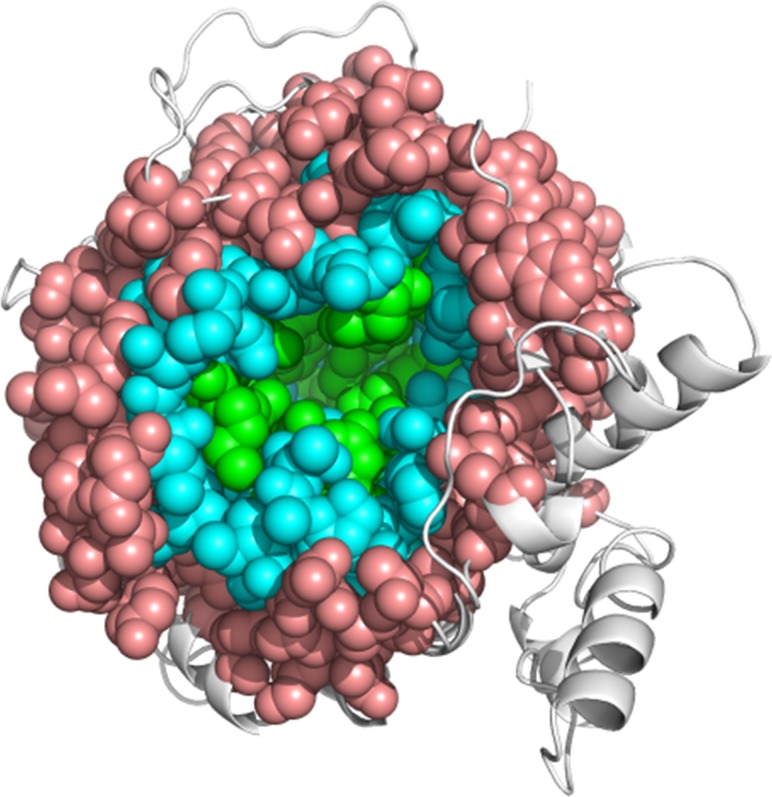
Distribution of Sfβgly residues based on their distances from the active site. Active site residues (green) are directly involved in substrate binding (GBS and ABS) and cleavage (CR); Residues composing the Layer 1 (L1, cyan) surround the active site and directly contact functional residues; Residues composing the Layer 2 (L2, salmon) envelop L1 and indirectly contact the active site *via* L1 (cyan) residues.

Using the solved Sfβgly structure, we firstly mapped amino acids that directly contact each functional residue from CR, GBS and ABS (S4 Fig). These residues form a contiguous external “shell” around the functional residues of the active site (Fig 6). We designate this first external shell of residues as Layer 1 (L1), as illustrated in cyan in Fig 6. We hypothesised that a mutation of an L1 residue would perturb the corresponding contacted functional region and reproduce effects similar to those observed for mutations within that particular functional region (ABS, GBS or CR) of the active site. Therefore, changes in L1 residues contacting GBS and CR residues would result in significant decreases in hydrolytic activity, while mutations in L1 residues contacting ABS would cause a broader range of effects. All mutations in L1 residues directly contacting the GBS (T35, W54, W143, P188, N400 and F460) are detrimental to Sfβgly catalytic efficiency (Table 1). Mutation T35A produced the least pronounced decrease (5-fold), which results from a negative modulation of *k*_cat_, (Table G in S1 File), while all others cause drastic decreases on Sfβgly hydrolytic activity when using NPβglc as substrate (71- to 24,000-fold, Fig 5, Table 1) due to a strong negative modulation of both the affinity for NPβglc and the catalytic rate (Table G in S1 File). Similar mutational effects are also observed for assays using NPβfuc as substrate (Table F in S1 File). Mutations of L1 residues exclusively contacting CR are also harmful: mutations F98A and T398A inactivate Sfβgly, and S378G decreases the Sfβgly hydrolysis rates of NPβglc and NPβfuc in 8- and 14-fold, respectively (Fig 5; Table 1 and Table F in S1 File). It is interesting to note that the mutant S378G also present a positive modulation of the *K*_m_, but a strong negative modulation of the *k*_cat_, as previously observed for the direct change of residues from CR (Table G in S1 File). The only exception seems to be the newly described mutant Y420A, which despite presenting enhanced hydrolytic activity using both NPβglc and NPβfuc as substrates (Fig 5; Table 1 and Table F in S1 File) also shows decreased hydrolytic activity towards its natural substrate cellobiose (see below). Finally, mutations of L1 residues exclusively contacting the ABS cause a wide range of mutational effects: modifications in G195, Y196 and P203 reduced Sfβgly hydrolytic activity using both synthetic substrates, while the M57A mutant had only a moderate effect (an approximately 1.3-fold decrease for both substrates) and the F334A mutant presented a 3-fold increase in catalytic efficiency using NPβglc as substrate. The harmful effects seen for mutations in L1 residues contacting the ABS (less than 17-fold decreases) are less pronounced than those observed for mutations in L1 contacting GBS or CR residues (Fig 5). Moreover, mutations in L1 that enhance the Sfβgly hydrolysis rates are mostly contacting the ABS. For example, mutations in residues R189, S247 and N249 that contact both ABS and CR result in catalytic enhancement (Fig 5) because they are located in the vicinity of the substrate aglycone (S3 Fig). However, while mutation R189G increases *k*_cat_, the other positive L1 mutations (S247, N249 and F334) increase the enzyme affinity for the substrate (decreases in the *K*_m_) (Table G in S1 File). Finally, changes in L1 residues directly contacting only GBS and CR reproduce the deleterious effects observed for mutations within these two functional regions.

These findings motivated us to investigate whether residues located even more distant from the active site could coherently modulate the Sfβgly activity. We hypothesised that structural perturbations caused by mutations beyond L1 could be transmitted through L1 residues to the functional residues of the Sfβgly active site, modulating its activity in a predictable way. This idea is analogous to the fine-tuning modulation of activity in allosteric enzymes, in which the information flows between allosteric and active sites through contact paths formed by amino acid interacting between them [40,41].

Analysis of the Sfβgly crystal structure allowed us to map amino acids surrounding L1, which likewise form a contiguous shell of amino acids here called layer 2 (L2; Fig 6, salmon). L2 residues therefore indirectly interact with the active site residues *via* L1. The mutational effects of nine positions located in L2 were correlated with the functional regions they indirectly contact through the shortest pathway (S4 Fig). It is worth noting that the more distant one residue is from the active site, the more possible paths it has to reach functional regions. As a direct consequence, most of the characterized L2 residues indirectly contact more than one functional region. Thus, effect of mutations in L2 that interact with more than one functional region would be the balance of effects on the activity dependent on each perturbed functional region.

Mutations indirectly contacting only the GBS (R474A and R474H) greatly reduce hydrolytic activity of Sfβgly using both NPβglc and NPβfuc as substrates, as well as for mutations in L2 residues indirectly contacting both GBS and CR (D84A, H223A and S424F) or both GBS and ABS (P62A) (Fig 5; Table 1 and Table F in S1 File). Therefore, all of the mutants in L2 residues that indirectly contact the GBS are deleterious to Sfβgly activity, mainly affecting the catalysis rate. When analysing L2 residues that indirectly contact only the ABS, we observed that the L350A mutation increases the enzyme affinity for NPβglc (Table G in S1 File), leading to a increase in more than 2.5-fold the Sfβgly hydrolysis of both synthetic substrates, while mutations S358F and S358A cause less than 3-fold decreases in hydrolytic rates (reductions that are less drastic than those observed L2 mutants contacting GBS) (Fig 5, Table 1 and Table F in S1 File). Similarly, mutation F251A, which indirectly contacts both ABS and CR, but not the GBS, causes a mild decrease in the hydrolytic rate (less than 2-fold) for both substrates (Fig 5; Table 1 and Table F in S1 File). Lastly, mutation K366A, which indirectly contacts all the three functional regions, increases the Sfβgly hydrolytic activity towards NPβglc and NPβfuc, contrasting with the deleterious perturbations observed for other mutations reaching the GBS. However, this contradiction can be explained by noting that K366 indirectly perturb the GBS through contacts between its main chain and L1 residue W402 (S5 Fig), whereas its side chain does not contact any GBS residue. Conversely, the K366 side chain contacts the ABS through its neighbouring L1 residue T373 (L1). Therefore, perturbations in the K366A side-chain would be expected to perturb more profoundly the neighbourhood of the ABS than the GBS. In fact, K366 increases the Sfβgly affinity for NPβglc, similarly observed for other mutations of residues related to the ABS (Table G in S1 File). The dataset of beneficial mutants suggest that increases in the Sfβgly catalytic efficiency is achieved through decreases in *K*_m_ values (increased affinity by the substrate).

Mutations in L1 and L2 residues that simultaneously perturb CR and GBS (S4 Fig) decrease the Sfβgly hydrolytic rates to degrees similar to those observed for perturbations at GBS residues. Analogously, L1 and L2 mutations simultaneously perturbing CR and ABS (R189, S247, N249 and F251) are less detrimental or even beneficial to the Sfβgly catalytic efficiency, reproducing the effects observed for mutations of ABS residues. Thus, external mutations simultaneously perturbing CR and a second functional sector (GBS or ABS) are likely to reproduce the effects observed for the latter. This indicates that CR is less sensitive to short- (L1) and mid-range (L2) perturbations than GBS and ABS.

Overall, our data strongly support a model in which L1 and L2 residues modulate Sfβgly activity through their contacts with the active site residues. The effects of mutations that originate outside the active site are transmitted to its functional residues and reproduce the effects observed for the direct mutations of the corresponding functional residues.

### Improving the cellobiase activity of β-glycosidases

Some mutations perturbing the ABS increase the Sfβgly hydrolysis rates towards the synthetic substrate NPβglc. However, even though NPβglc presents the same glycone motif as cellobiose (a natural substrate), their aglycone motifs are different. As a consequence, important interactions between the active site and different substrates are distinct, and activity enhancements observed for synthetic substrates may not result in the enhanced hydrolysis of natural ones, as cellobiose. Thus, in order to correlate the enhancement of the hydrolytic activity towards synthetic and natural substrates, we determined the kinetic parameters for the hydrolysis of cellobiose for the eight new Sfβgly mutants characterized in this study (Table 2). As control for negative effects, we also tested mutants R97A, located within the CR, and S358A, which has previously demonstrated moderate negative effects using synthetic substrates [9].

**Table 2:** Kinetics of mutational effects for the hydrolysis of cellobiose (*k*_cat_/*K*_m_). The relative *k*_cat_/*K*_m_ corresponds to [(*k*_cat_/*K*_m_)_mut_/(*k*_cat_/*K*_m_)_WT_].

Mutation	*K*_m_ (mM)	*k*_cat_ (s^-1^)	*k*_cat_/*K*_m_ (cellobiose as substrate)	Relative *k*_cat_/*K*_m_
**WT**	**2.36**	**2.61**	**1.11**	**-**
D84A*	**-**	**-**	**0.0125**	**0.011**
R97A*	**-**	**-**	**0.0014**	**0.0013**
**S247A**	**3.32**	**2.89**	**0.87**	**0.79**
**N249A**	**1.3**	**7.52**	**5.78**	**5.23**
**F251A**	**1.04**	**0.74**	**0.71**	**0.64**
**F334A**	**0.33**	**1.08**	**3.22**	**2.91**
**L350A**	**4.51**	**3.31**	**0.73**	**0.66**
**S358A**	**0.93**	**0.09**	**0.094**	**0.085**
**K366A**	**1.63**	**1.65**	**0.98**	**0.89**
**Y420A**	**3.1**	**0.99**	**0.32**	**0.29**

* Mutants D84A and R97A present linear increase of activity due to increase of substrate concentration, and *k*_cat_/*K*_m_ values were determined using the curve slope and assuming that used substrate concentrations were very low when compared to the enzymes *K*_m_ (pseudo-first order kinetic).

As expected, the R97A mutant decreases Sfβgly hydrolytic activity towards the hydrolysis of cellobiose in more than 700-fold. Deleterious effects on Sfβgly cellobiase activity were also observed for mutations D84A and S358A. Both of these positions are located in L2, the first indirectly contacting the GBS while the second indirectly contacts the ABS. The moderate negative effect observed for mutation F251A using synthetic substrates was reproduced in assays using cellobiose as substrate. Still, mutations S247A, L350A, K366A and Y420 result in mild reductions in Sfβgly catalytic efficiency. Although mild, these observed decrements are distinct from the increased hydrolytic activity observed for Sfβgly when using synthetic substrates. However, the low detrimental intensity of those decreases (including the observed for F251A) has close to neutral effects to Sfβgly hydrolytic activity. On the other hand, mutants N249A and F334A increase the cellobiase activity of Sfβgly, reproducing the increments in activity observed when using synthetic substrates. Both mutations have increased affinity for cellobiose, but while the former also presents increased *k*_cat_ values, the latter presents a decreased in the catalytic constant. Still, although mutants F251 and K366 present a slight decrease in activity, they increase the Sfβgly affinity for cellobiose.

Given that most of mutations introduced in enzymes found in nature produce deleterious or neutral effects [42], the findings described here that some mutants positively enhance activity is not usual for hydrolases. For instance, saturation mutagenesis in the TEM-1 β-Lactamase only yielded mutants with equal or reduced efficiency when compared to the wild-type enzyme [43]. However, in this case neutral mutations were shown to be important for the evolution of one mutant with a new function (resistance to cefotaxime) from the original TEM-1 β-Lactamase. Thus, the finding of several positions associated to the ABS likely to accept variability is very promising: three mutations are neutral (M57, F251 and M453) and seven enhance the hydrolysis rate of Sfβgly towards NPβglc, with similar results observed for some of these mutants using cellobiose as substrate. These positions are good starting points from which to introduce variability aiming to obtain more efficient GH1 β-glycosidases, which agrees with the previous suggestion to target residues in the first and second “shells” of amino acids surrounding the active site within a distance up to 12 Å from key active site residues [44]. Our beneficial mutations are also supported by similar results achieved by mutating ABS residues in the Rice BGlu1 [12], for which mutation in L442 (corresponding to the Sfβgly M453) is neutral using cellobiose and NPβglc as substrates, while mutations in I179, N190 and N245 (corresponding to the Sfβgly E190, K201 and N249, positions also described here) enhance the enzymatic efficiency of cellobiose hydrolysis [12]. Moreover, beneficial mutations (L167W, P172L and F179H) also have been described for the *Trichoderma reesei* β-glycosidase (TrBgl2) using NPβglc as substrates [45]. The mutation P172L, which yields the highest increase in TrBgl2 activity, corresponds to the previously described Sfβgly position E194 [11], and thus is located inside the ABS. The same is also true for TrBgl2 mutations L167W and F179H, which also correspond to ABS positions R189 [9] and K201 [11] in Sfβgly, whose mutations increase the Sfβgly hydrolytic activity towards NPβglc. Therefore, these findings also support that perturbations in the ABS are the responsible for increasing the hydrolytic activity in β-glycosidases.

In this sense, the rational design of more efficient β-glycosidases for the hydrolysis of cellobiose should initially target positions inside or adjacent to the ABS, for which positive effects are observed, and avoid perturbations inside or adjacent to the GBS and CR, which mostly yield deleterious mutants and is supported by mutations of CR and GBS residues in the β-glycosidases from *N*. *koshunensis* [19] and the fungus *Trichoderma reesei* TrBgl2 [45]. We anticipate as hotspots for future mutagenesis studies the positions corresponding to R189, K201, S247, N249, F251, F334, L350, K366 and M453 (Sfβgly numbering), which are located in the vicinity of substrate aglycone (S3 Fig; Fig 4) and whose mutations enhance the hydrolysis rate of synthetic substrates in Sfβgly, with similar results observed in the TrBgl2 [45] and in Rice Bglu1 [12].

Despite the fact that Sfβgly, TrBgl2 and Rice BGlu1 are not as catalytically efficient as commercial enzymes for cellobiose degradation, the correlations observed for mutants of these enzymes from diverse taxa (animals, fungi and plants) suggest the existence of preferential positions to be targeted in GH1 β-glycosidases aiming to produce faster enzymes. For instance, the hydrolytic activity of commercial β-glycosidases (e.g. from *Aspergillus sp*.) could be further enhanced by the introduction of combinatorial mutagenesis into positions corresponding to those here described. Furthermore, given the high structural conservation among GH1 β-glycosidases, these mutations could be introduced in thermo-resistant enzymes, such as the β-glycosidases from *Pyrococcus furiosus* (PDB ID 3APG) [46] and *T*. *neapolitana* [39].

Lastly, it is noteworthy that the large majority of mutations analysed here replaced the native amino acid residue with alanine. In some cases, we observed opposing effects depending on the specific structural change introduced at that position (i.e. K201A *vs* K201F and R189A *vs* R189G). Therefore, replacements for other amino acids than Alanine at the predicted hotspots listed above could yield even greater increments than the ones we observed and could further improve the hydrolytic activity of other β-glycosidases.

## Supporting Information

S1 Fig Comparisons among β-glycosidase dimers. A-B—Surface representation of rystallographic dimers. A: Sfβgly (Chain A: Green; Chain B: Red); B: *Neotermes koshunensis* β-glycosidase dimer (PDB: 3AHZ) formed between monomers belonging to different unit cells. C–Cartoon representation of crystallographic contacts (sticks in yellow and green) predicted by PDBePISA [32] for *N*. *koshunensis* β-glycosidase. D–Dimers superposition between Sfβgly (green) and β-glycosidase from *Brevicoryne brassicae* (pink; PDB: 1WCG).
(TIF)

S2 Fig Surface representation of the active site entrance in Sfβgly. The active site is located above the β-barrel (cartoon) and functional residues from GBS (Red), ABS (blue) and CR (Yellow) are in sticks. One Tris molecule bound in the active site (sticks in green) denotes the active site entrance. Note that ABS residues are located in the active site opening (surface in blue), while the GBS is placed on the bottom of the active site (surface in red).
(TIF)

S3 Fig Sfβgly active site complexed with cellobiose (sticks in magenta). Transparent residues in sticks: active site GBS (red), ABS (blue) and CR (yellow) residues; beneficial mutations: green sticks. Residues R189, S247, N249 and F334, whose mutations increased Sfβgly activity, are close to the aglycone portion of cellobiose, thus these positions are also close to the ABS (sticks in blue) but distant from GBS (sticks in red).
(TIF)

S4 Fig Mutations in Sfβgly grouped by functional regions they perturb. A: Intersections contain positions relatd to more than one functional region. Active site residues are in italics; residues from layer 1 are underlined; residues from layer 2 are double underlined. B: Heat mapping of contacts determined from the crystallographic structure of Sfβgly. Residues closer than 5 Å were considered as a contact. Lemon green highlights the lowest number of steps to reach the active site (1 step means direct contact to active site residues, i.e. L1). Active site residues (top of the table) are colored according to their functional region: red, GBS; yellow, CR; blue, ABS.
(TIF)

S5 Fig Structural explanation why mutation K366A perturbs the ABS but not the GBS. The K366 backbone contacts W402, and the replacement K366A would not perturb the GBS residue W444 (Red). However, the K366 side chain contacts T373, and the mutation K366A would perturb this interaction, which could be transmitted to the ABS residue W371 (Blue).
(TIF)

S1 File This file contains all Supporting Tables (1–7). (PDF)

## Abbreviations

GH1: glycoside hydrolase family 1
Sfβgly: β-glycosidase from *Spodoptera frugiperda*
CR: Catalysis-related residues
GBS: Glycone-binding site
ABS: Aglycone-binding site
PDB: Protein Data Bank
NPβglc: *p*-nitrophenyl-β-D-glucopyranoside
NPβfuc: *p*-nitrophenyl-β-D-fucopyranoside
MUβglc: methylumbellyferyl β-D-glucoside
L1/L2: residues at Layer 1/2 surrounding the active site residues
