# Supplementary figures and images for: Using the Amino Acid Network to Modulate the Hydrolytic Activity of β-Glycosidases
Source: PLoS One. 2016 Dec 9;11(12):e0167978. doi: 10.1371/journal.pone.0167978 (PMC5148593; doi:10.1371/journal.pone.0167978)

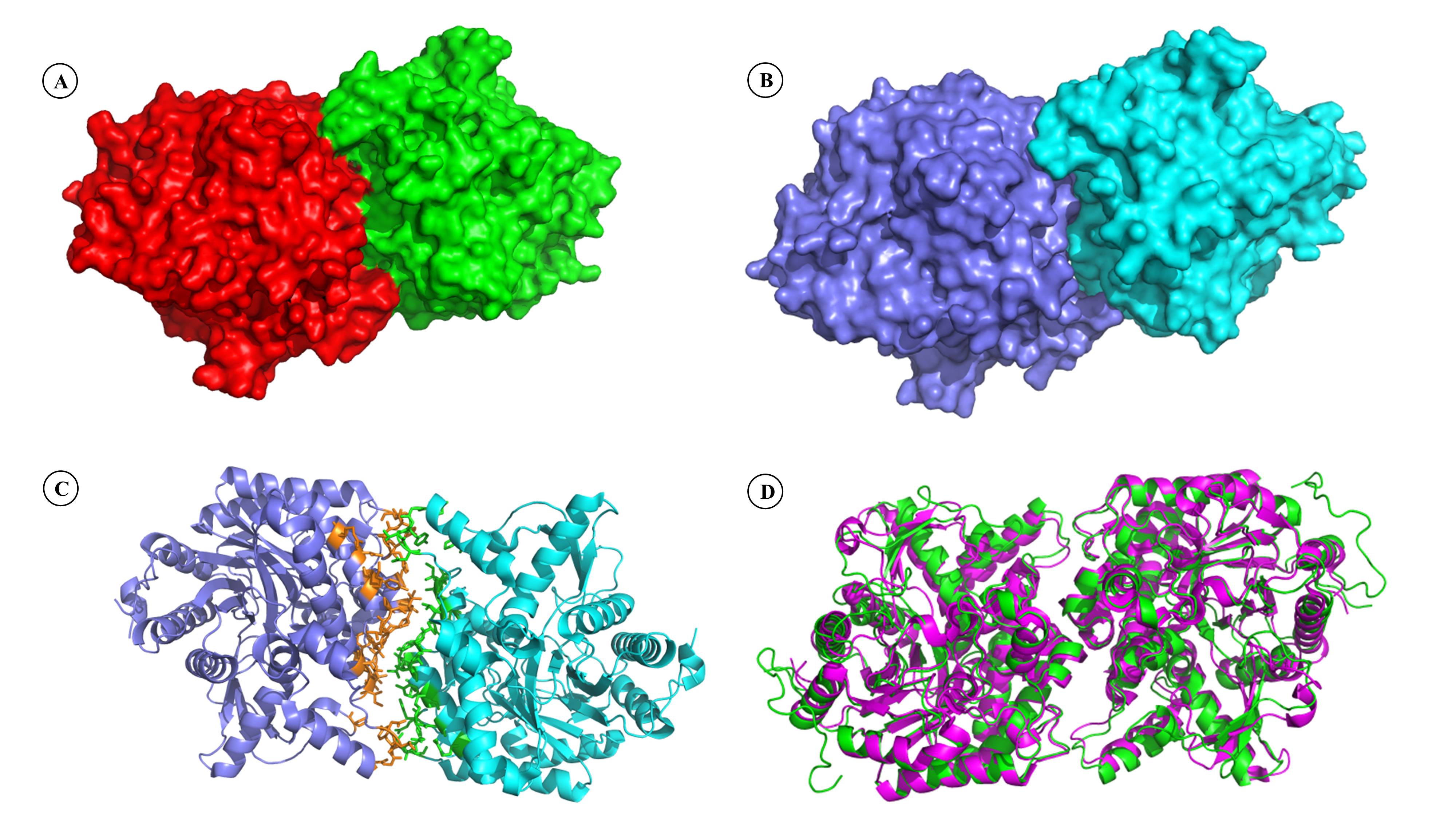

Supplement: S1 Fig — A-B—Surface representation of rystallographic dimers. A: Sfβgly (Chain A: Green; Chain B: Red); B: Neotermes koshunensis β-glycosidase dimer (PDB: 3AHZ) formed between monomers belonging to different unit cells. C–Cartoon representation of crystallographic contacts (sticks in yellow and green) predicted by PDBePISA [32] for N. koshunensis β-glycosidase. D–Dimers superposition between Sfβgly (green) and β-glycosidase from Brevicoryne brassicae (pink; PDB: 1WCG). (TIF) [file pone.0167978.s001.TIF]

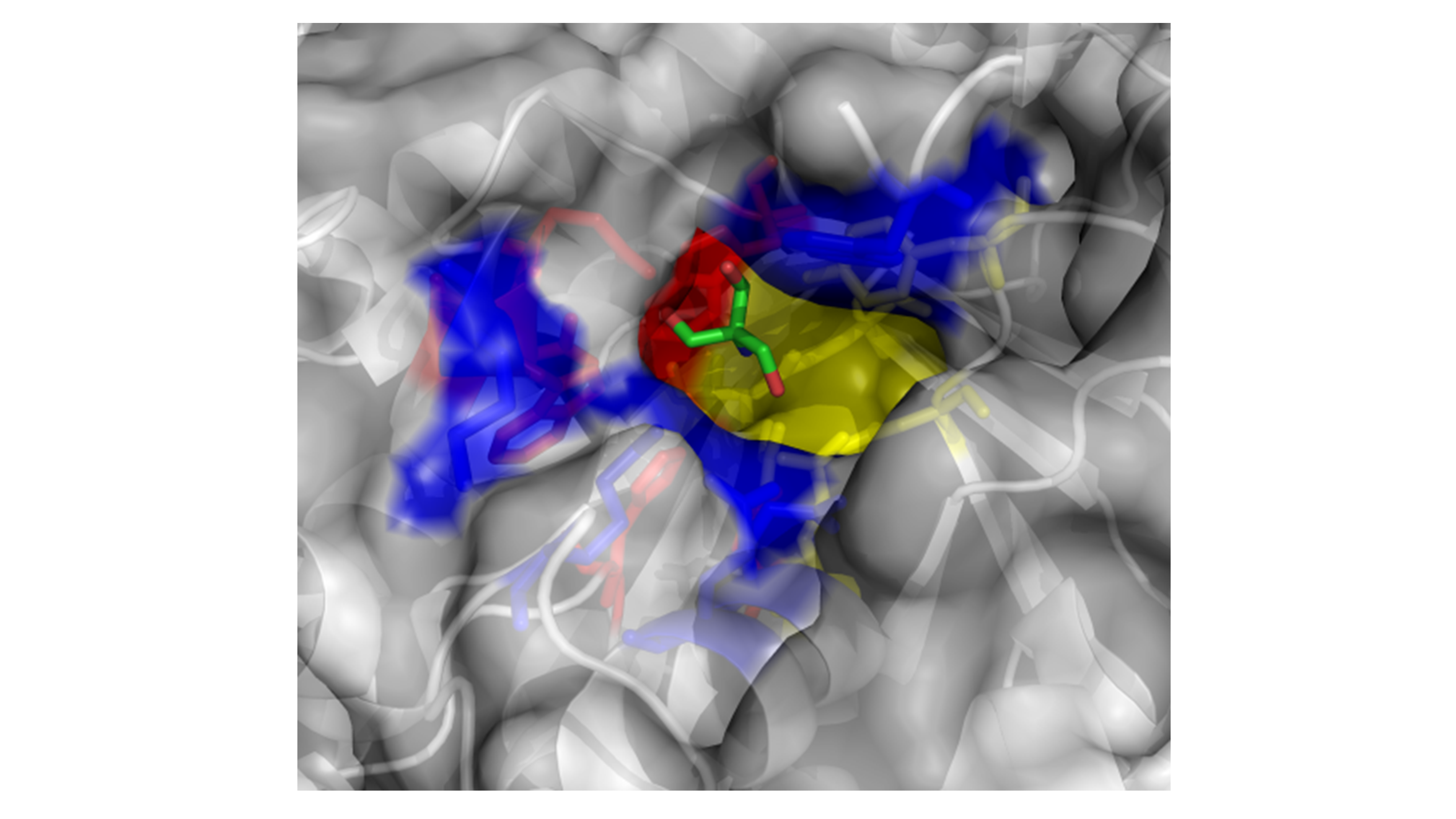

Supplement: S2 Fig — The active site is located above the β-barrel (cartoon) and functional residues from GBS (Red), ABS (blue) and CR (Yellow) are in sticks. One Tris molecule bound in the active site (sticks in green) denotes the active site entrance. Note that ABS residues are located in the active site opening (surface in blue), while the GBS is placed on the bottom of the active site (surface in red). (TIF) [file pone.0167978.s002.TIF]

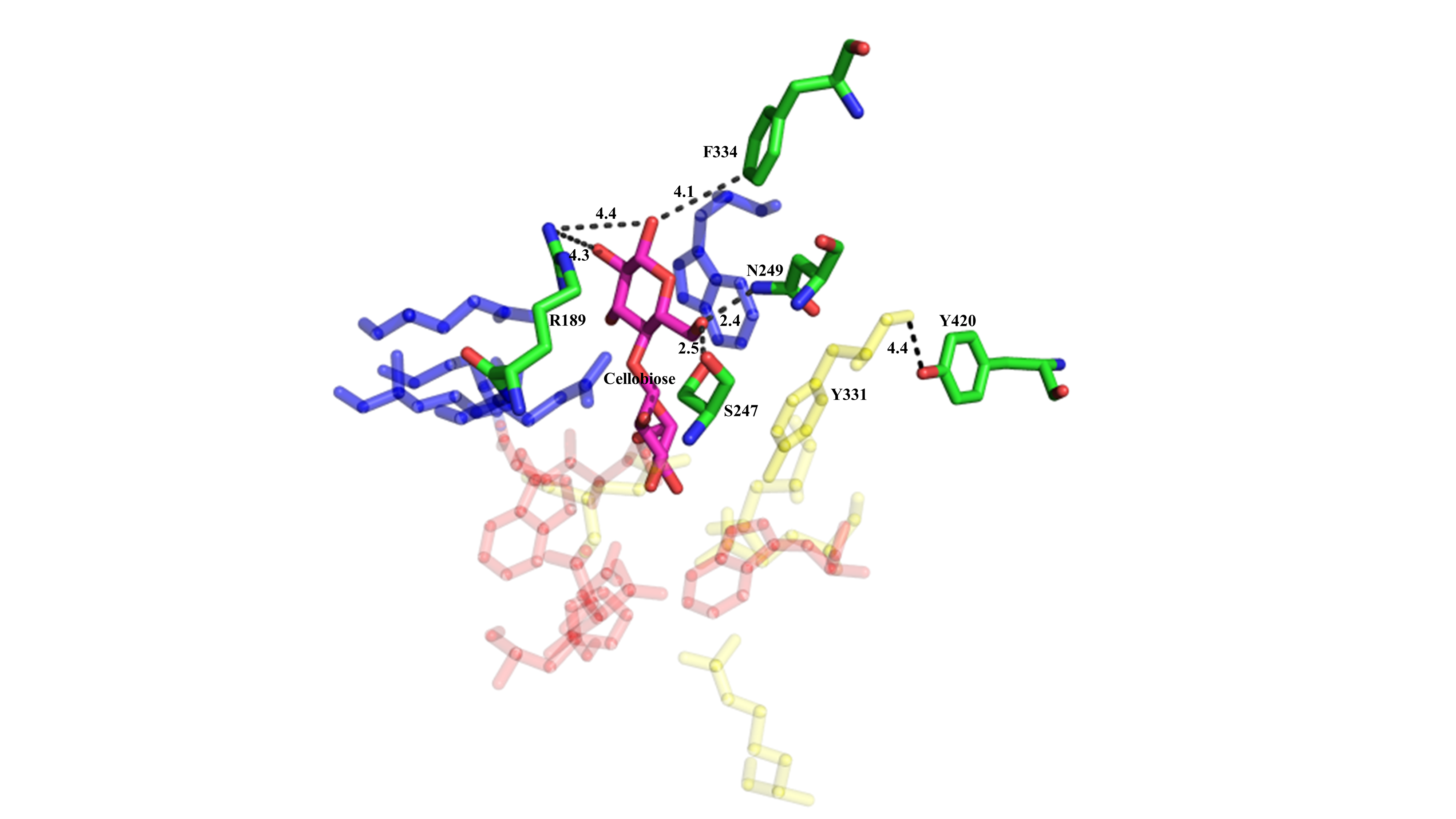

Supplement: S3 Fig — Transparent residues in sticks: active site GBS (red), ABS (blue) and CR (yellow) residues; beneficial mutations: green sticks. Residues R189, S247, N249 and F334, whose mutations increased Sfβgly activity, are close to the aglycone portion of cellobiose, thus these positions are also close to the ABS (sticks in blue) but distant from GBS (sticks in red). (TIF) [file pone.0167978.s003.TIF]

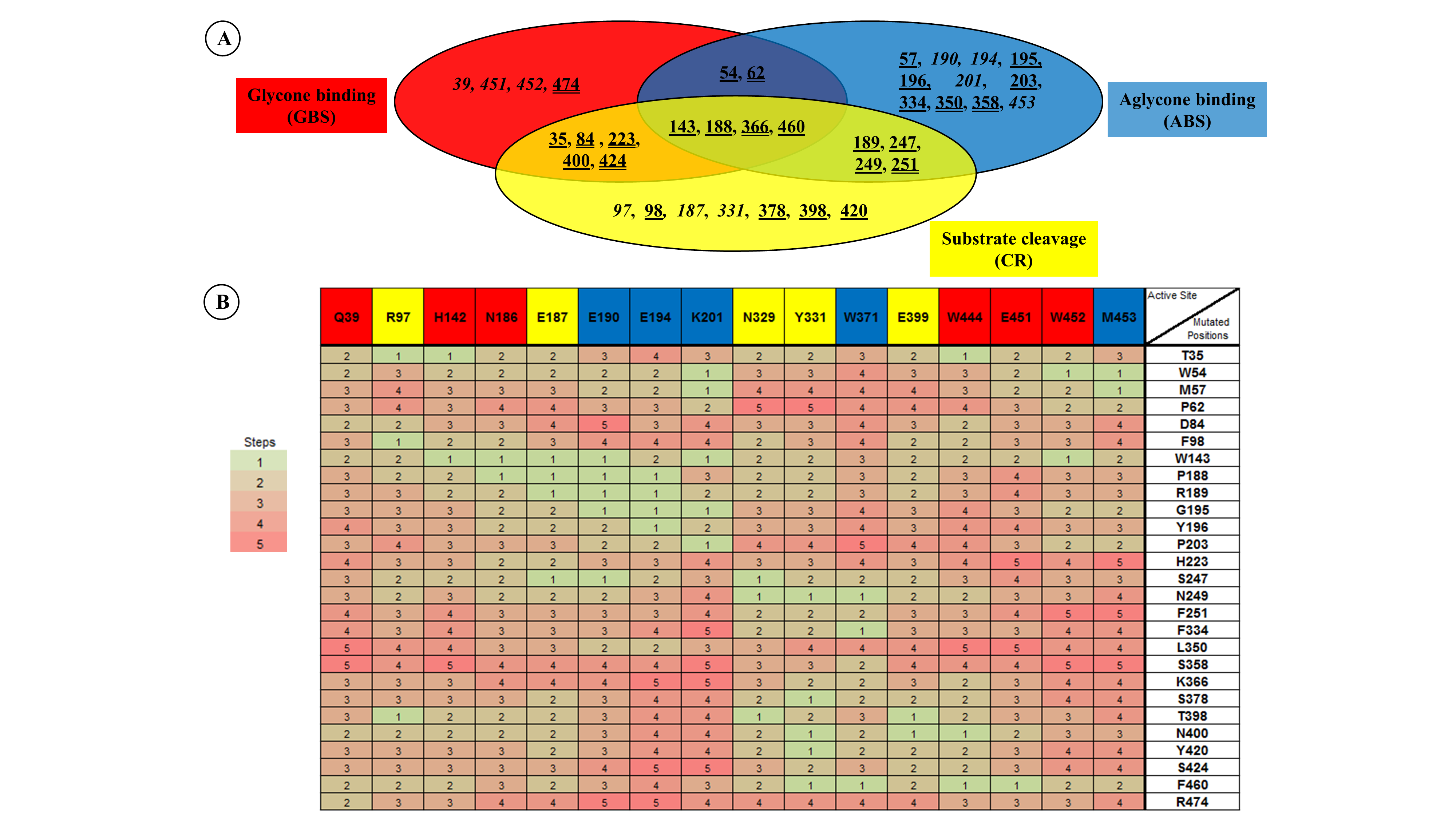

Supplement: S4 Fig — A: Intersections contain positions relatd to more than one functional region. Active site residues are in italics; residues from layer 1 are underlined; residues from layer 2 are double underlined. B: Heat mapping of contacts determined from the crystallographic structure of Sfβgly. Residues closer than 5 Å were considered as a contact. Lemon green highlights the lowest number of steps to reach the active site (1 step means direct contact to active site residues, i.e. L1). Active site residues (top of the table) are colored according to their functional region: red, GBS; yellow, CR; blue, ABS. (TIF) [file pone.0167978.s004.TIF]

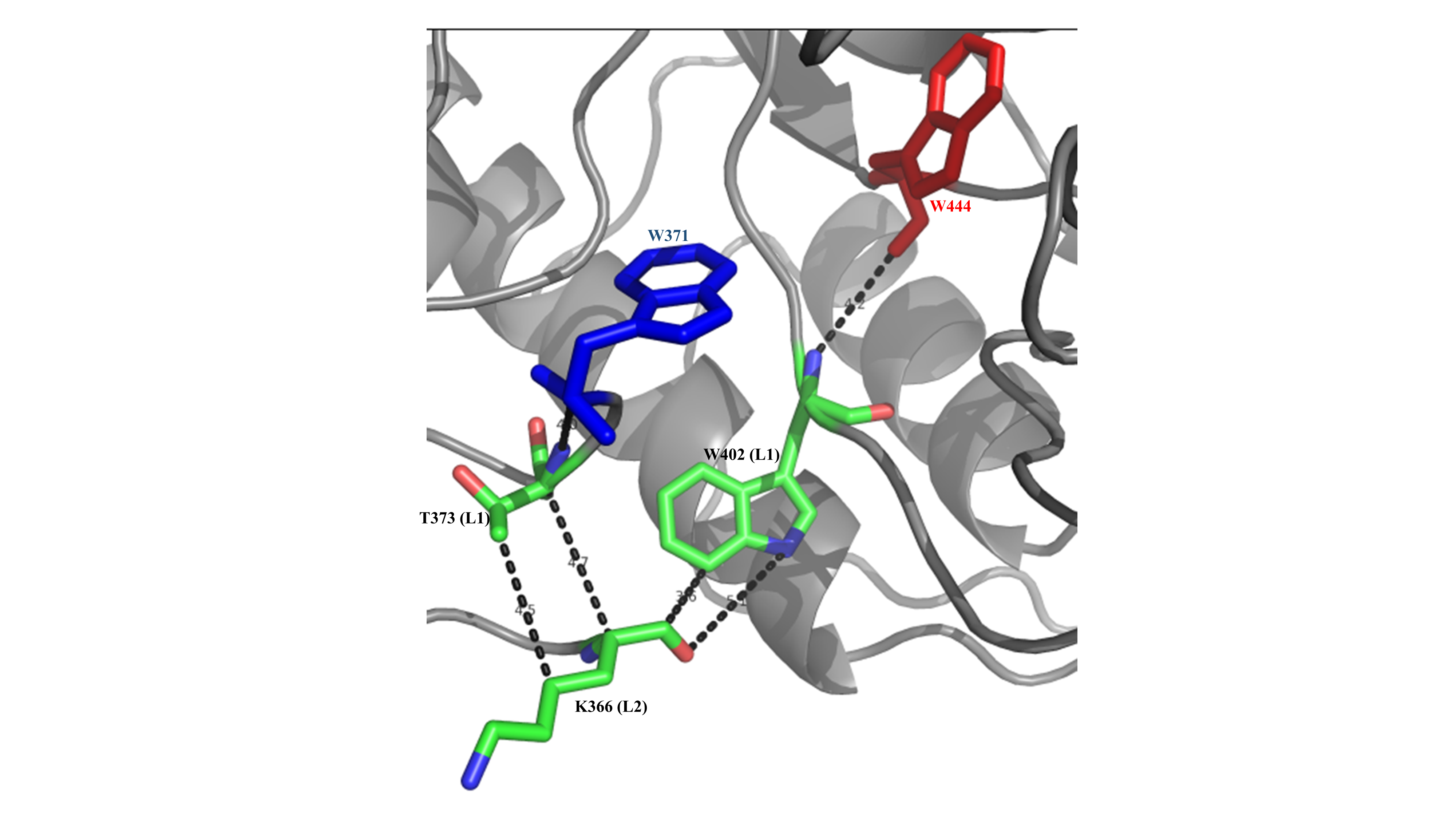

Supplement: S5 Fig — The K366 backbone contacts W402, and the replacement K366A would not perturb the GBS residue W444 (Red). However, the K366 side chain contacts T373, and the mutation K366A would perturb this interaction, which could be transmitted to the ABS residue W371 (Blue). (TIF) [file pone.0167978.s005.TIF]
