# Supplementary material for: Using the Amino Acid Network to Modulate the Hydrolytic Activity of β-Glycosidases
Source: PLoS One. 2016 Dec 9;11(12):e0167978. doi: 10.1371/journal.pone.0167978 (PMC5148593; doi:10.1371/journal.pone.0167978)
Supplement: S1 File — (PDF) [file pone.0167978.s006.pdf]

SUPPLEMENTARY MATERIAL

Table A. Mutagenic primers for site directed mutagenesis.

Mutation	Primer sequence
**D84A FW**	5’ ccacaactacaagcgtgctgtcgagatgatgagg 3’
**D84A RV**	5’ cctcatcatctcgacagcacgcttgtagttgtgg 3’
**R97A FW**	5’ ggcctggacgcttacgcgttctccctctcctgg 3’
**R97A RV**	3’ ccaggagagggagaacgcgtaagcgtccaggcc 5’
**S247A FW**	5’ cagtgcggtattaccatcgcugtaaactggtttggaccag 3’
**S247A RV**	3’ gtcacgccataatggtagcgacatttgaccaaacctggtc 5’
**N249A FW**	5’ gcggtattaccatcagtgtagcctggtttggaccagcccactcc 3’
**N249A RV**	3’ cgccataatggtagtcacatcggaccaaacctggtcgggtgagg 5’
**F251A FW**	5’ ccatcagtgtaaactgggctggaccagccacgccccac 3’
**F251A RV**	3’ ggtagtcacatttgacccgacctggtcggtgcggggtg 5’
**F334A FW**	5’ gaaccactacacagcagccctggtatcggcgactgaac 3’
**F334A RV**	3’ cttggtgatgtgtcgtcgggaccatagccgctgacttg 5’
**L350A FW**	5’ cgtaccctgtgccatctgcactggatgatgtggacac 3’
**F350A RV**	5’ gtgtccacatcatccagtgcagatggcacagggtacg 5’
**S358A FW**	5’ gatgatgtggacactggcgcctgggctgatgatagctg 3’
**S358A RV**	3’ ctactacacctgtgaccgcggacccgactactatcgac 5’
**Y420A FW**	5’ gatgatgacaggatccaggcctacaggcttccatgg 3’
**Y420A RV**	3’ ctactactgtcctaggtccggatgtcccgaaggtacc 5’

Table B. Data collection and refinement statistics for Sfβgly.

**Data Collection**	
Wavelength (Å)	1.5419
Space group	P1
Cell dimensions	
a, *b*, *c* (Å)	53.83, 64.99, 257.19
α, β, γ (°)	93.16, 92.04 , 112.18
Resolution (Å)	49.75 – 2.09 (2.18 – 2.09)
No. of measured reflections	566,584
No. of unique reflections	173,155
Data Completeness (%)	91.0 (91.1)
Redundancy	3.3 (3.2)
$I/\sigma(I)$	12.5 (1.8)
R_{merge}	0.080 (0.539)
R_{meas}	0.095 (0.356)
R_{pim}	0.050 (0.356)
$CC_{1/2}$	(0.709)
**Refinement**	
Protein molecules per asymmetric unit	6
Total number of protein residues<br>Protein chains A/B/C/D/E/F	2906<br>488/488/488/486/485/471
Total number of non-hydrogen atoms	25,131
No. of protein atoms	23,639
No. of ligand atoms	174
No. of water molecules	1,318
Resolution (Å)	49.75 – 2.09 (2.18 – 2.09)
No. of reflections	164,477
R factor	0.192 (0.344)
R_{free}	0.250 (0.356)
Root-mean-square deviations from ideal values	
Bond lengths (Å)	0.008
Bond angles (°)	1.199
Average B values (Å^2)	43.0
Protein all atoms	41.0
Protein chains A/B/C/D/E/F	32.8/36.8/36.6/47.5/50.3/55.3
Water	44.2
Tris	69.2
NAG	77.1
Ramachandran plot analysis (%)	
Residues in favored regions	2754 - 94.83%
Residues in allowed regions	148 - 5.03%
Residues in disallowed regions	4 - 0.14%

Values in parentheses refer to data in the highest-resolution shell. R_{free} is calculated based on 5% of the reflections.

Table C. RMSD values (Å) for structural alignments between different Sfβgly chains and between each Sfβgly chain and a previously used homology model [14].

Sfβgly chain	B	C	D	E	F	Homology model
A	0.092	0.084	0.118	0.139	0.107	0.839
B	X	0.091	0.115	0.137	0.143	0.833
C		X	0.115	0.145	0.098	0.827
D			X	0.192	0.137	0.880
E				X	0.141	0.775
F					X	0.834

RMSD values were automatically calculated for aligned atoms by using PyMOL structural alignments.

Table D. RMSD values (Å) for structural alignments between Sfβgly (chain A) and homologous β-glycosidase structures from PDB (ID codes shown).

PDB code	3AI0	1E6S	1E4I	1E56	1UG6	2ZOX	1V03	1VFF
Sfβgly chain A	0.571	0.795	0.757	0.824	0.68	0.757	0.744	1.046

RMSD values were automatically calculated for aligned atoms by using PyMOL structural alignments.

Table E. Residue-residue pairing involved in dimer contacts predicted by DCA.

Residue 1	Residue 2	DI value	Ranking position (total 111,156)
152	163	0.1336951	39
156	211	0.0796443	102
157	163	0.0461175	249
110	152	0.0448486	264
111	163	0.0336649	488
112	150	0.0310610	615

Table F. Effects of single mutations over the k_{cat}/K_m for the hydrolysis of NPβfuc. Residues are separated by each functional region of the Sfβgly active site they contact.

Glycone Binding (GBS)		Aglycone Binding (ABS)		Substrate Cleavage (CR)	
Mutation	Relative k_{cat}/K_m	Mutation	Relative k_{cat}/K_m	Mutation	Relative k_{cat}/K_m
T35A[1]	0.33	W54A[4]	0.025	T35A[1]	0.33
Q39A[2]	0.00036	M57A[4]	0.827	D84A	inactive
Q39E[3]	0.096	P62A[4]	0.19	*R97A*	0.00001
Q39N[3]	0.012	W143A[4]	0.001	*R97M*[1]	0.021
W54A[4]	0.025	P188A[4]	0.001	F98A[4]	inactive
P62A[4]	0.19	R189G[1]	1.8	W143A[4]	0.001
D84A	inactive	R189A[1]	0.004	*E187D*[6]	inactive
W143A[4]	0.001	*E190A*[5]	0.193	P188A[4]	0.001
P188A[4]	0.001	*E190Q*[5]	0.333	R189G[1]	1.8
H223A[2]	0.236	*E194A*[5]	0.833	R189A[1]	0.004
K366A	1.6	G195L[4]	0.037	H223A[4]	0.236
N400D[1]	0.005	Y196A[4]	0.069	S247A	2.8
N400A[1]	0.005	*K201A*[5]	1.1	N249A	3.0
N400V[1]	0.017	*K201F*[5]	4.6	F251A	0.63
S424F[1]	0.006	P203A[4]	0.23	*Y331F*[1]	0.004
E451A[2]	0.000059	S247A	2.8	K366A	1.6
E451Q[3]	0.13	N249A	3.0	S378G[1]	0.072
E451D[3]	0.014	F251A	0.63	T398A[4]	Inactive
E451S[3]	0.004	F334A	0.9	N400D[1]	0.005
W452A[4]	inactive	L350A	2.5	N400A[1]	0.005
F460A[4]	0.005	S358F[1]	0.26	N400V[1]	0.017
F460L[1]	0.026	K366A	1.6	Y420A	2.3
R474H[1]	0.036	*M453A*[5]	2.1	S424F[1]	0.006
R474A[1]	0.002	F460A[4]	0.005		
		F460L[1]	0.026		

Relative k_{cat}/K_m corresponds to $[(k_{cat}/K_m)_{mut}/(k_{cat}/K_m)_{WT}]$. Mutations that cause relative k_{cat}/K_m decrement higher or lower than 4 fold are respectively marked in yellow and light purple; mutations that increase the relative k_{cat}/K_m are in green. Residues belonging to layer 1 are underlined; residues from layer 2 are double underlined; active site residues are in italics. Kinetics from mutations D84A, R97A, S247A, N249A, F251A, F334A, L350A, K366A and Y420A are new data here presented. 1: Mendonça and Marana, 2011 [9]; 2: Marana *et al.*, 2002 [7]; 3: Marana *et al.*, 2004 [8]; 4: Tamaki *et al.*, 2014 [14]; 5: Mendonça and Marana, 2008 [11]; 6: Marana *et al.*, 2003 [6]. For calculation of the Relative k_{cat}/K_m each $k_{cat}/K_{m\ mut}$ was compared to the k_{cat}/K_{mWT} data presented on the same manuscript in which the mutant enzyme was firstly described 1: Mendonça and Marana, 2011 [9]; 2: Marana *et al.*, 2002 [7]; 3: Marana *et al.*, 2004 [8]; 4: Tamaki *et al.*, 2014 [14]; 5: Mendonça and Marana, 2008 [11]; 6: Marana *et al.*, 2003 [6].

Table G. Variation of k_{cat} and K_m by functional region of the Sfβgly active site. Values are the ratio between mutant and wild-type kinetic parameters using NPβglc as substrate. K_m variation ($[K_m]_{mut}$ / $[K_m]_{WT}$) values higher than 1 indicates that mutations decrease the affinity for NPβglc, and k_{cat} variation ($[k_{cat}]_{mut}$ / $[k_{cat}]_{WT}$) values higher than 1 indicates that a mutation increases the catalytic rate towards the substrate NPβglc.

Glycone Binding (GBS)			Aglycone Binding (ABS)			Substrate Cleavage (CR)		
Mutation	K_m Variation	k_{cat} Variation	Mutation	K_m Variation	k_{cat} Variation	Mutation	K_m Variation	k_{cat} Variation
T35A[1]	1.02	0.22	W54A[4]	1.78	0.04	T35A[1]	1.02	0.22
Q39A[2]	5.1	0.0006	M57A[4]	0.68	0.53	D84A	-	-
Q39E[3]	3.6	0.047	P62A[4]	0.58	0.023	*R97A*	1.2	0.000009
Q39N[3]	13.3	0.0084	W143A[4]	4.9	0.0018	*R97M*[1]	1.2	0.017
W54A[4]	1.78	0.04	P188A[4]	N.D.	N.D.	F98A[4]	-	-
P62A[4]	0.58	0.023	R189G[1]	1.2	4.1	W143A[4]	4.9	0.0018
D84A	-	-	R189A[1]	5.4	0.023	*E187D*[6*]	1.9	0.00085
W143A[4]	4.9	0.0018	*E190A*[5]	1.2	0.26	P188A[4]	N.D.	N.D.
P188A[4]	N.D.	N.D.	*E190Q*[5]	2.3	0.29	R189G[1]	1.2	4.1
H223A[2]	1.4	0.035	*E194A*[5]	1.3	0.25	R189A[1]	5.4	0.023
K366A	0.56	1.23	G195L[4]	0.51	0.06	H223A[4]	1.4	0.035
N400D[1]	4.3	0.011	Y196A[4]	2.5	0.16	S247A	0.196	0.67
N400A[1]	1.35	0.0037	*K201A*[5]	2.9	0.29	N249A	0.174	0.59
N400V[1]	2.3	0.032	*K201F*[5]	1.3	1.8	F251A	0.52	0.29
S424F[1]	3.2	0.13	P203A[4]	12.2	0.74	*Y331F*[1]	0.14	0.0023
E451A[2]	6.9	0.000075	S247A	0.20	0.67	K366A	0.56	1.23
E451Q[3]	3.3	0.13	N249A	0.17	0.59	S378G[1]	0.73	0.10
E451D[3]	3.8	0.0072	F251A	0.52	0.29	T398A[4]	-	-
E451S[3]	5.56	0.00056	F334A	0.48	1.25	N400D[1]	4.3	0.011
W452A[4]	-	-	L350A	0.43	1.31	N400A[1]	1.35	0.0037
F460A[4]	14.1	0.019	S358F[1]	1.16	0.42	N400V[1]	2.3	0.032
F460L[1]	3.51	0.024	S358A[1]	1.54	0.89	Y420A	0.56	0.90
R474H[1]	2.3	0.013	K366A	0.56	1.23	S424F[1]	3.2	0.13
R474A[1]	0.34	0.0045	*M453A*[5]	1.4	0.67			
			F460A[4]	14.1	0.019			
			F460L[1]	3.51	0.024			

For calculation of the variations each k_{cat} and K_m of the mutant enzymes were compared to the k_{cat} and K_m of the wild-type enzyme presented on the same manuscript in which the mutant enzyme was firstly described. Mendonça and Marana, 2011 [9] (K_m: 0.74 mM; k_{cat}: 0.19 s^{-1}); 2: Marana *et al.*, 2002 [7] (K_m: 0.45 mM; k_{cat}: 2.4 s^{-1}); 3: Marana *et al.*, 2004 [8] (K_m: 0.45 mM; k_{cat}: 2.4 s^{-1}); 4: Tamaki *et al.*, 2014 [14] (K_m: 4.1 mM; k_{cat}: 0.70 s^{-1}); 5: Mendonça and Marana, 2008 [11] (K_m: 0.90 mM; k_{cat}: 2.26 s^{-1}); 6: Marana *et al.*, 2003 [6] (K_m: 2.3 mM; k_{cat}: 1.73 s^{-1}; *: This mutant was studied using MUβglc as substrate); This paper: (K_m: 2.3 mM; k_{cat}: 0.61 s^{-1})
